# Small Bowel Imaging from Stepchild of Roentgenology to MR Enterography, Part II: The Reliable Disclosure of Crohn’s Disease and Non-Inflammatory Small Bowel Disorder Plot through MRI Findings

**DOI:** 10.3390/life13091836

**Published:** 2023-08-30

**Authors:** Antonio Pierro, Laura Maria Minordi, Luigi Larosa, Carla Cipri, Giulia Guerri, Fabio Quinto, Fabio Rotondi, Annalisa Marcellino, Raffaella Basilico, Roberto Iezzi, Savino Cilla

**Affiliations:** 1 Radiology Unit, San Timoteo Hospital, 86039 Termoli, Italy; antonio.pierro@asrem.org; 2 Radiology Unit, Fondazione Policlinico Universitario A. Gemelli IRCCS, 00168 Roma, Italy; lauramaria.minordi@policlinicogemelli.it (L.M.M.); luigi.larosa@policlinicogemelli.it (L.L.); carla.cipri@policlinicogemelli.it (C.C.); giulia.guerri@policlinicogemelli.it (G.G.); roberto.iezzi@policlinicogemelli.it (R.I.); 3 Angiography Unit, “L. Bonomo” Hospital, 70031 Andria, Italy; quintofabio@libero.it; 4 Oncological Surgery Unit, Responsible Research Hospital, 86100 Campobasso, Italy; fabio.rotondi@gemellimolise.it; 5 ASReM, Azienda Sanitaria Regionale del Molise, Via Ugo Petrella 1, 86100 Campobasso, Italy; annalisa.marcellino@asrem.org; 6 Department of Neurosciences, Imaging and Clinical Studies, “Gabriele D’Annunzio” University, 66100 Chieti, Italy; raffaella.basilico@asl2abruzzo.it; 7 Medical Physics Unit, Responsible Research Hospital, 86100 Campobasso, Italy

**Keywords:** small bowel, MR enterography, Crohn’s disease, bowel wall enhancement patterns

## Abstract

MRE has become a standard imaging test for evaluating patients with small bowel pathology, but a rigorous methodology for describing and interpreting the pathological findings is mandatory. Strictures, abscess, inflammatory activity, sinus tract, wall edema, fistula, mucosal lesions, strictures, and mesentery fat hypertrophy are all indicators of small bowel damage in inflammatory and non-inflammatory small bowel disease, and they are all commonly and accurately explained by MRE. MRE is a non-invasive modality that accurately assesses the intra-luminal, parietal, and extra-luminal small bowel. Differential MRE appearance allows us to distinguish between Crohn’s disease and non-inflammatory small bowel disorder. The purpose of this paper is to present the MRE pathological findings of small bowel disorder.

## 1. Introduction

Crohn’s disease (CD) is a chronic granulomatous inflammatory disorder of unknown etiology, characterized by transmural intestinal inflammation. The terminal ileum and the caecum are the most affected areas of the digestive tract, even though it can occur in any region of the digestive system. Typically, in CD the inflammatory areas are multiple, and they are clearly separated from healthy tissue at both macroscopic and microscopic level, giving rise to the so-called skip lesions.

Imaging has an undisputed role in the diagnosis of CD and in the evaluation of the disease after treatment. Furthermore, MRE has a great impact considering that it is possible to comprehensively assess the small bowel for pathology and classify it according to extent, severity, and complications, as well as to assess their response to therapy. According to ESGE [1] and ECCO [2] guidelines, MRE is recommended to investigate the extent of CD and extraluminal involvement in patients with a known diagnosis of CD. On the contrary, capsule endoscopy is the favored initial diagnostic option for evaluating the small intestine in patients with suspected CD and a negative ileocolonoscopic examination in the absence of obstructive symptoms or radiological stenosis.

In order to accurately estimate the precise disease burden through the imaging manifestations, radiologists have to provide a rigorous methodology for describing and interpreting the pathological findings.

Our review aims to provide an overview of the current status of small bowel imaging, with an emphasis on MR enterography. The historical, technical, and clinical underpinnings of these small bowel imaging methods were discussed in part 1 of this review [3]. 

Here in part 2, we focus on current and emerging clinical applications of MR enterography in Crohn’s disease and other small bowel disorders. The several opportunities and challenges in the clinical translation and deployment of small bowel imaging in routine clinical practice are presented. With continued progress in this active field, MR enterography in Crohn’s disease is expected to have an increasing role in our understanding of small bowel diseases.

## 2. MR Enterography in Crohn’s Disease Affecting the Small Bowel: A Guide for Observation and Interpretation of Imaging Findings

The MRE signs of CD have been extensively investigated and meticulously described over the years [4,5,6,7,8]. To help gastroenterologists and intestinal surgeons make the appropriate management decisions, radiologists must interpret imaging findings considering their clinical implications. Indeed, owing to an appropriate description and interpretation of MRE imaging it is possible to follow the right therapeutic road. Because some findings can be shown in various pathologies or have different implications, therefore it is essential to describe all the manifestations (e.g., location and distribution) in the specific clinical scenario to avoid misinterpretation.

**Figure 1:**
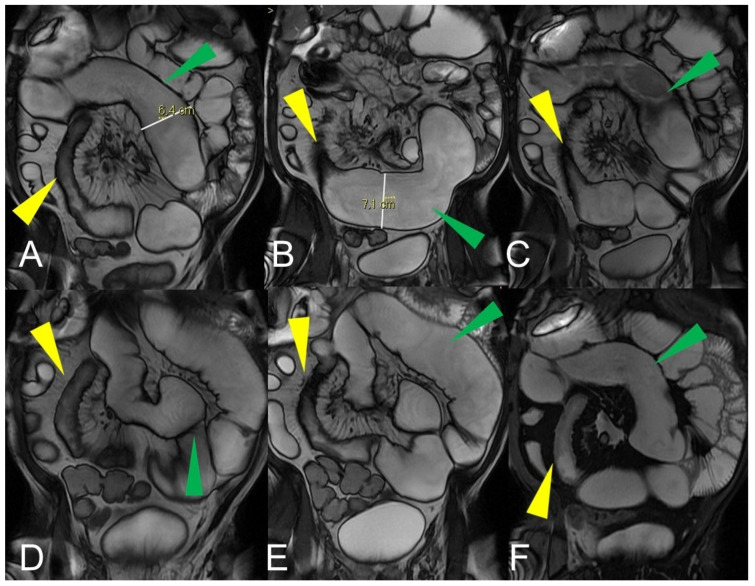
Quantification of intestinal strictures. Coronal fast imaging employing steady-state acquisition (FIESTA) images (**A**–**F**) show a long segment of the distal ileum affected by pathology (yellow arrowheads) showing, reduction in caliber, stiffness, and absent peristalsis. The upstream loops are extremely dilated (green arrowheads) indicating that, although a tight stenosis of the lumen is not appreciable, the long segment reduced in caliber behaves as if it were a pre-occlusive stenosis, preventing the transit of intestinal contents with massive dilatation of the upstream loops. This condition represents a functional stenosis that needs to be treated surgically to prevent repeated subocclusions or intestinal obstruction or other complications.

**Figure 2:**
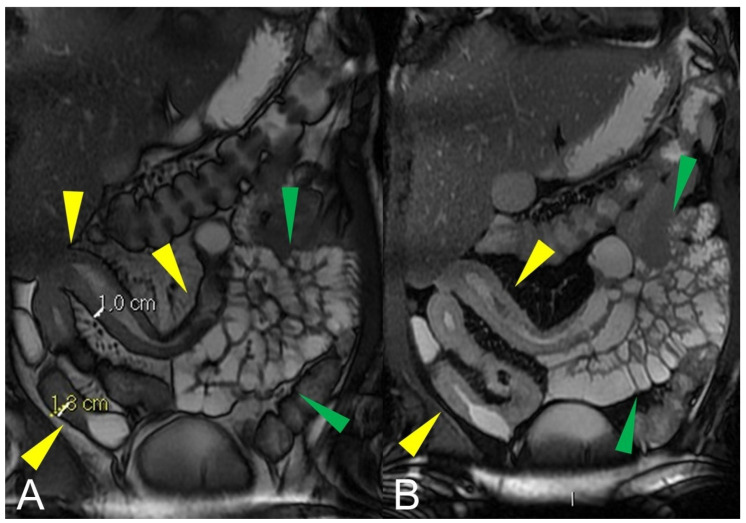
Quantification of intestinal strictures. Coronal fast imaging employing steady-state acquisition (FIESTA) images (**A**,**B**) show a long segment of the distal ileum affected by pathology (yellow arrowheads). The upstream loops do not show dilatation (green arrowheads) because the pathological loops do not show significant abnormal stiffness or massive luminal reduction. This condition represents a no-functional “stenosis” because the intestinal flow is preserved, so there is no risk of sub-occlusions or intestinal obstruction.

Bruining et al. [6] well described this known and anecdotal crucial point reporting the differences between a long and a short rigid stenosis in terms of therapeutic approach and clinical relevance. In fact, relative moderate bowel stenosis distributed over a long segment behaves as a short but pre-occlusive stenosis, preventing intestinal flow and resulting in the upstream dilation of the normal loops. This is defined as a functional stenosis that will probably lead to bowel obstruction whereby a conservative non-surgical treatment would be fallacious. Therefore, it is necessary to know the location of the pathological tissue influencing the medical or surgical therapeutic choices [6] (Figure 1 and Figure 2).

The three great fields in the CD are (a) bowel wall manifestations, (b) penetrating disease, and (c) mesenteric findings. They represent the macroscopic manifestation of the chronic transmural inflammatory condition that potentially leads to structural damage (bowel damage or organ damage). 

### 2.1. Bowel Wall in Crohn’s Disease

**Figure 3:**
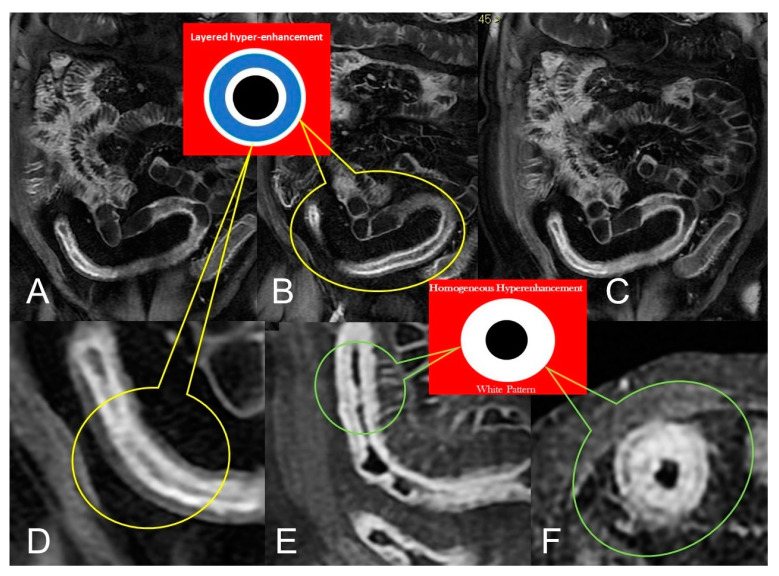
Active inflammatory Crohn’s disease with layered hyper-enhancement pattern (**A**–**D**): the presence of active Crohn’s disease is indicated by small bowel wall thickening with layered hyperenhancement on coronal fat-suppressed contrast-Enhanced T1-Weighted MRE images. Active inflammatory Crohn’s disease with homogeneous enhancement pattern (**E**,**F**): the presence of active Crohn’s disease is indicated by small bowel wall thickening with homogeneous enhancement on coronal (**E**) and axial (**F**) fat-suppressed contrast-Enhanced T1-Weighted MRE images.

**Figure 4:**
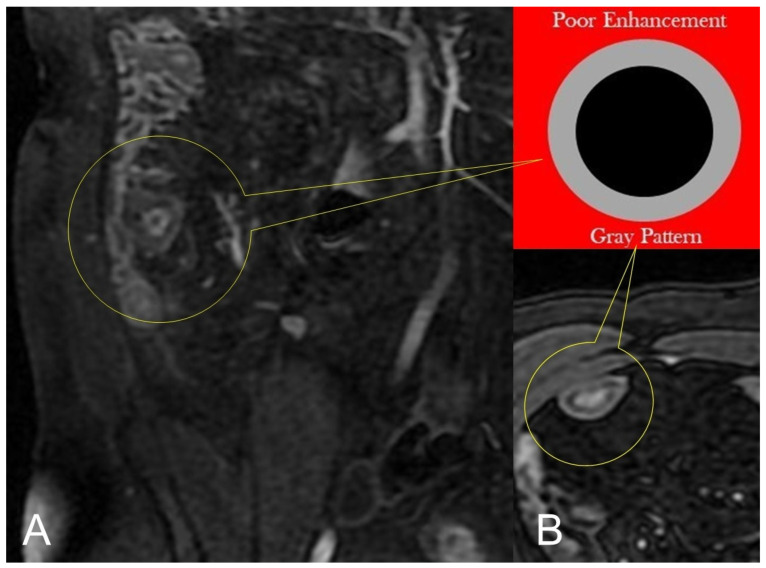
Lack of layered or homogeneous enhancement can be related to fat ((**B**), axial FIESTA) image, or fibrosis parietal deposition, with poor enhancement and gray pattern on contrast-enhanced fat-suppressed T1-weighted image ((**A**), coronal image).

In CD, segmental bowel wall mural hyperenhancement represents the pathological tissue that at contrast-enhanced MRE is revealed as increased mural signal intensity in a small bowel’s segment compared with the normal adjacent intestinal tract [5]. Mural hyperenhancement can be asymmetric (in a small bowel loop, it could involve the mesenteric border more than the antimesenteric one), stratified (bilaminar or trilaminar inner-wall hyperenhancement or halo sign), or homogeneous (evenly distributed over the entire bowel wall). Submucosal edema, granulation tissue, intramural fat accumulation, fibrosis, or inflammatory infiltration may contribute to stratified enhancement (Figure 3 and Figure 4). Bowel wall enhancement evaluation is correctly depicted in the enteric phase (45–50 s after the intravenous contrast material injection begins) and/or in portal venous phase (60–70 s after the intravenous contrast material injection begins). Furthermore, we consider also that endoscopy and histopathological examination clearly show the absence of mucosa in the affected intestinal segments, so the term “mucosal hyperenhancement” is incorrect when the stratified enhancement pattern is expressed and should not be used. Finally, it must be underlined that the segmental bowel wall mural hyperenhancement is a sensitive but non-specific sign of CD, and therefore it must always be contextualized in the general framework of interpretation [6].

**Figure 5:**
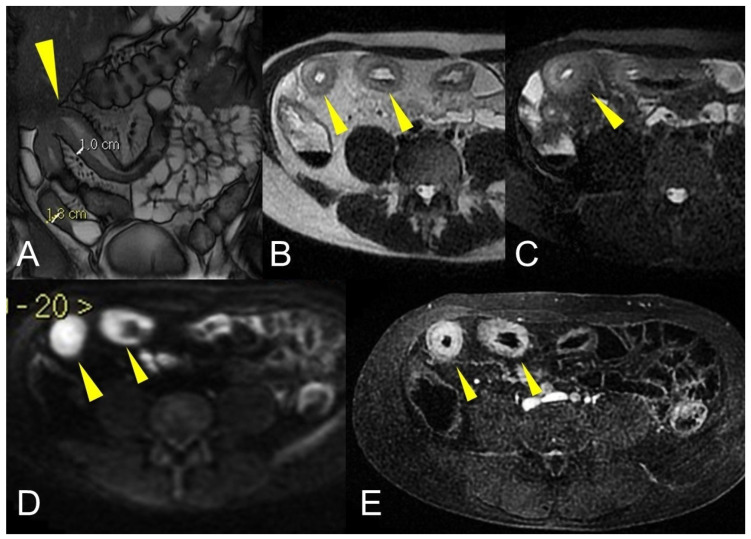
Active inflammatory Crohn’s disease component revealed by MRE imaging. Coronal fast imaging employing steady-state acquisition (FIESTA) image (**A**) show mural thickening involving the terminal ileum (yellow arrowhead). Axial T2-weighted MRE image (**B**) of the same patient shows small bowel hyperintense wall thickening (yellow arrowheads) with matching parietal edema, restricted diffusion, and homogeneous enhancement, respectively in axial T2-weighted images with fat suppression ((**C**), yellow arrowheads), axial diffusion-weighted image ((**D**), yellow arrowheads), and contrast-enhanced fat-suppressed T1-weighted image ((**E**), yellow arrowheads).

**Figure 6:**
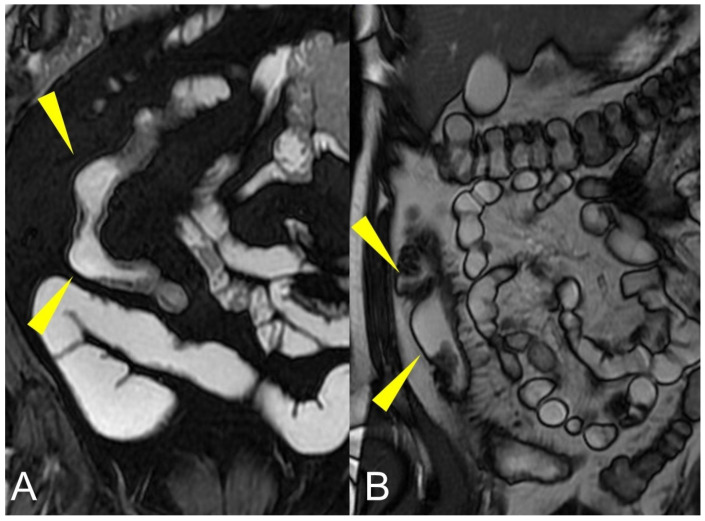
Coronal fast imaging employing steady-state acquisition (FIESTA) images (**A**,**B**) show sacculations or pseudodiverticular extroversions of the antimesenteric side of the affected intestinal loops due to due to acute or chronic mesenteric border inflammation (yellow arrowheads in (**A**,**B**)).

To assess the wall thickness, a good bowel distention is required (Figure 5A). Depending on the size, wall thickening should be subdivided as mild (3–5 mm), moderate (>5–9 mm), and severe (≥10 mm) [5]. However, asymmetric mass-like bowel wall thickening greater than 15 mm is atypical for Crohn’s disease, and cancer should be suspected. Intramural edema (Figure 5C) is typically investigated in fat-suppressed T2-weighted images where it appears as hyperintense intramural bowel. Ulcerations are consistent with a defect confined to the bowel wall, containing air or enteric intraluminal contrast that is focused in the inflamed bowel wall without extraluminal spreading [5,6]. The affected intestinal loops can present sacculation or pseudodiverticular extroversions of the antimesenteric side due to acute or chronic mesenteric border inflammation [5,6] (Figure 6). Intestinal strictures are defined as abnormal narrowing of bowel lumen with unequivocal upstream loop’s dilation. It is a common complication of CD and, according to the pathogenetic pathway, may be subdivided into fibrotic, inflammatory, and mixed types [6,7,8,9]. Important information in the radiological report should be the location, length, and dilatation’s size of the loops upstream to the stenosis (without upstream dilation if the lumen is <3 cm, mild upstream dilation if it is between 3–4 cm, and moderate to severe in upstream lumen >4 cm [6]. In upstream dilation greater than 4 cm, radiologists should report the presence of small bowel obstruction. Surgical treatment plays an important role in managing the CD’s stricture, especially when endoscopy is contraindicated (e.g., the intestinal tract is not endoscopically accessible or high risk of endoscopic dilatation) [9] (Figure 1 and Figure 2). An active inflammation may be recognized by restricted diffusion (Figure 5D); although this is a non-specific sign, it can be helpful when the bowel is inadequately distended [10]. Therefore, the use of DWI and ADC sequences must be performed by an experienced radiologist to contextualize the findings in the set of the multiparametric examination.

### 2.2. Beyond Bowel Wall in Crohn’s Disease: Penetrating Disease and Mesenteric Inflammation

Crohn’s disease progressively involves the entire thickness of the bowel wall by an uncontrolled inflammation process. Approximately 20–30% of patients develop complications such as fistulas or abscesses at diagnosis.

**Figure 7:**
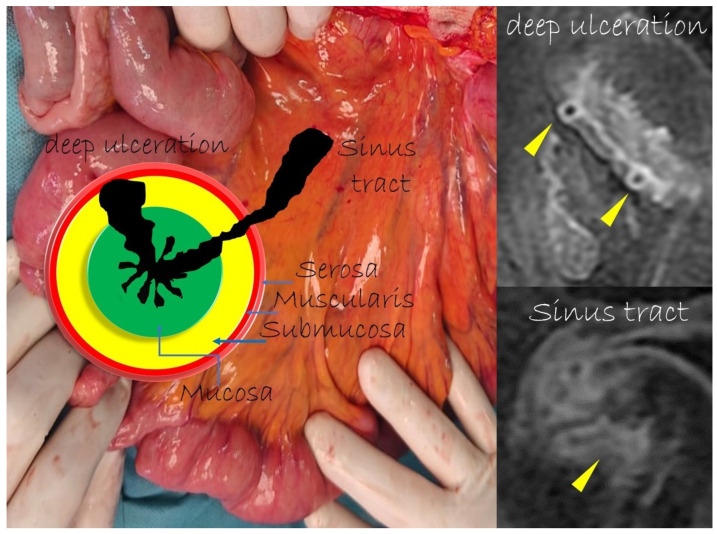
The sinus tracts that represent a sealed-off perforation that develops into a phlegmon or, if infected, into an abscess (9) but does not reach adjacent organs or the skin. Therefore, they end up blind.

**Figure 8:**
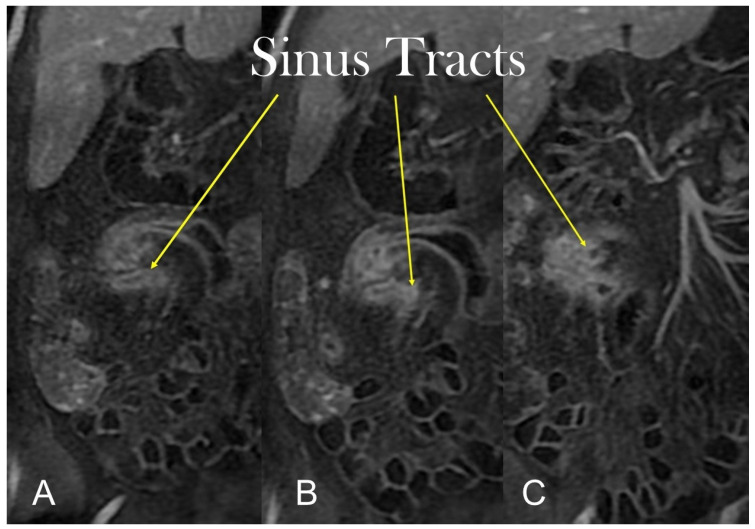
Coronal (**A**–**C**) contrast-enhanced fat-suppressed T1-weighted images of the same patient show the sinus tracts that represent a sealed-off perforation that develops into a phlegmon or, if infected, into an abscess (9) but does not reach adjacent organs or the skin. Therefore, they end up blind.

Transmural involvement can develop a sinus tract that reaches the serosa resulting in a fistula’s channel that connects different structures (e.g., entero-enteric, entero-vesical, and entero-vaginal fistula). In fact, sinus passages are closed off from perforations that result in phlegmons or, if infected, abscesses, but do not affect the nearby organs or the skin (Figure 7 and Figure 8).

**Figure 9:**
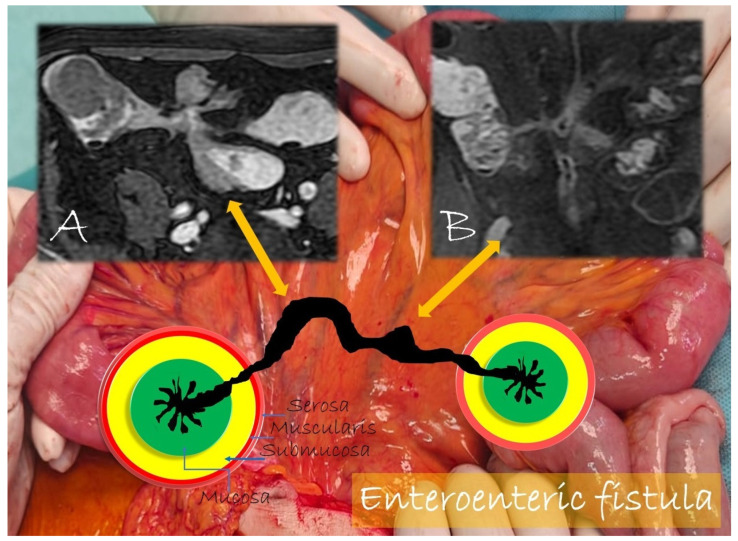
Pathological junctions of intestinal segments through enteroenteric fistulas, which represents internal fistulas that connect intestinal segments such as ileo-sigmoidal (**A**) and ileo-colic (**B**).

**Figure 10:**
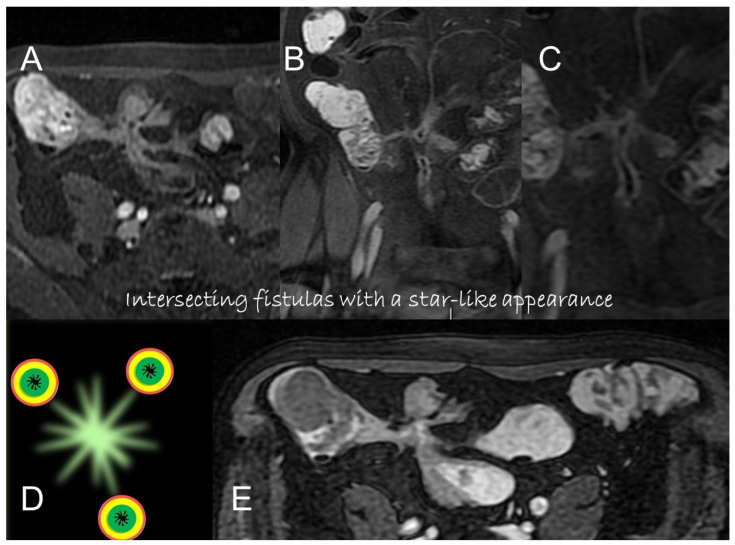
Axial (**A**) and coronal (**B**,**C**) contrast-enhanced fat-suppressed T1-weighted images and axial FIESTA image (**E**) of the same patient show a complex fistula formed by the intersection of multiple fistulous arms that connect pathological segments of the small intestine to each other and to the ascending colon through the mesentery. These fistulous pathways, intersecting each other, end up taking on the starry appearance, typical of complex fistulas (**D**).

**Figure 11:**
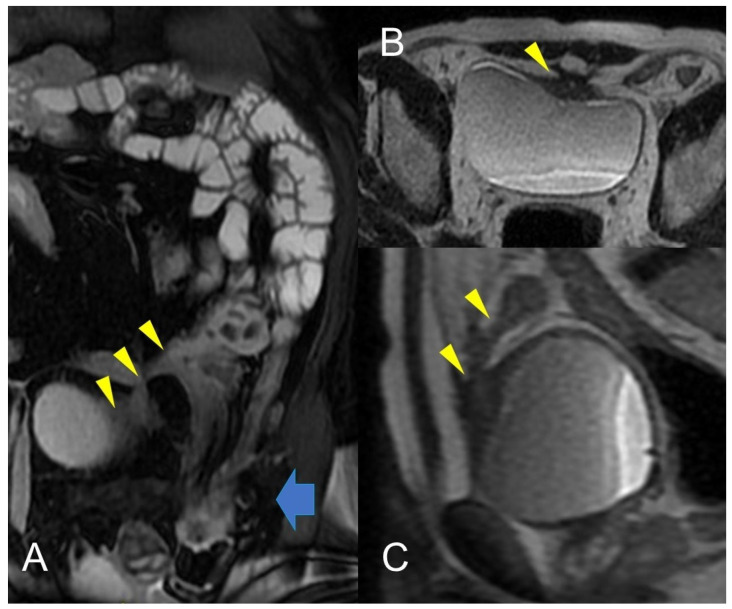
Crohn’s disease is a common cause of Enterovesical fistula formation. The enterovesical fistula represents an abnormal connection between the enteric lumen and the bladder. Coronal fast imaging employing steady-state acquisition (FIESTA) images (**A**) show a complex fistula with one arm consisting of an enterovesical fistula (yellow arrowheads). Axial (**B**) and sagittal (**C**) T2-weighted MRE image of the same patient shows enterovesical fistula. In (**A**), inguinal hernia indicated by the blue arrow with involvement of a pathological section of the small intestine.

Unlike the sinus tracts, a fistula is defined as a pathologic channel connecting two or more epithelialized surfaces [11]. Fistulae can be simple (single extra enteric tract: enter enteric, enter colic, enter vesical, enterocutaneous, or rectovaginal) or complex (branching and intersecting fistulas, sometimes with a star-like appearance [5]). They represent a complication in approximately 14–50% of the patients with Crohn’s disease (Figure 9, Figure 10 and Figure 11).

### 2.3. Inflammatory Mass or Inflammatory Conglomerate (Aka “Phlegmon”)

**Figure 12:**
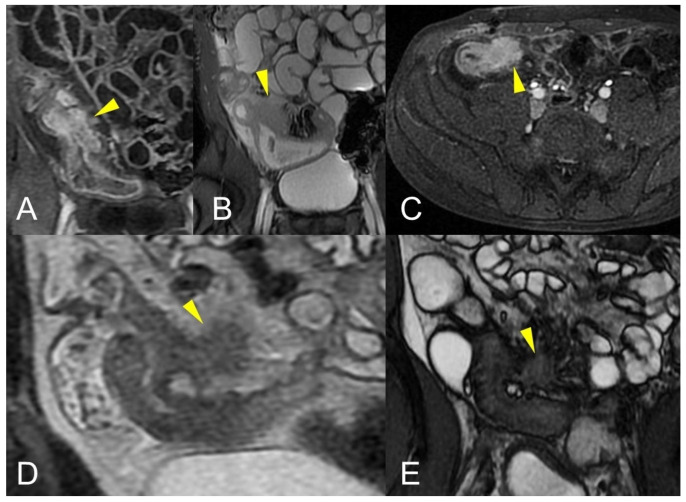
Inflammatory conglomerate represents a climbing mesenteric fat involvement in the context of penetrating disease and mesenteric inflammation. The components of the fistulas within the mass can be difficult to detect because it often coexists with phenomena of retraction that are expressed on the intestinal loops nearby or on the ureters. Small inflammatory conglomerate in the context of the mesentery adherent to a segment of the pathological small intestine, with active disease (yellow arrowheads in (**A**,**C**): coronal (**A**) and axial (**C**) contrast-enhanced fat-suppressed T1-weighted image and in (**B**,**E**) (coronal fast imaging employing steady-state acquisition; FIESTA image) and (**D**) (coronal T2-weighted MRE image)).

**Figure 13:**
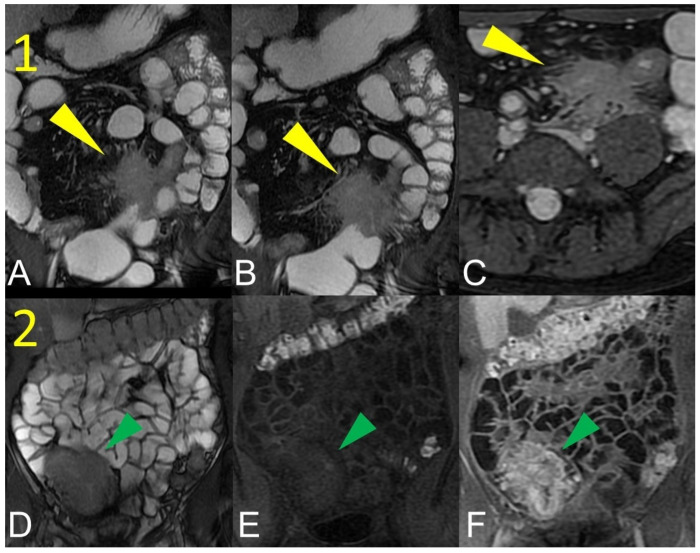
Two different patients with, respectively, an inflammatory conglomerate (Patient 1) of medium size (yellow arrowheads in (**A**–**C**)) and of large size (green arrowheads in (**D**–**F**)) (Patient 2). Coronal (**A**,**B**) and axial (**C**) fast imaging employing steady-state acquisition (FIESTA) image. Coronal FIESTA image (**D**) and coronal without (**E**) and with (**F**) contrast-enhanced fat-suppressed T1-weighted image. Notably intense contrast enhancement in (**F**) image underlines the significant state of inflammation of the disease in the active and complicated phase.

**Figure 14:**
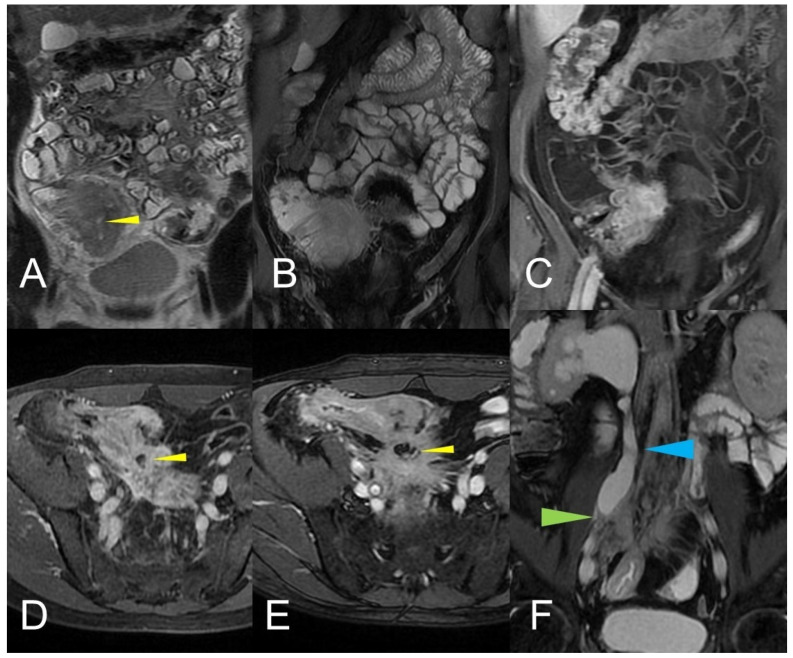
Large climbing inflammatory conglomerate. A small abscess is evident in the center of the inflammatory conglomerate (yellow arrowheads in (**A**,**D**,**E**)). It shows marked enhancement (yellow arrowhead in (**C**)) and determines conspicuous retracting effects both on the mesentery (spiculate aspect in (**D**,**E**)), in which it develops, and on the adjacent loops. In this patient, the right distal pelvic ureter is also narrowed, (green arrowhead in (**F**)) with hydronephrosis (blue arrowhead in (**F**)). (**A**): coronal T2-weighted MRE image; (**B**,**F**): coronal fast imaging employing steady-state acquisition (FIESTA) image; (**C**): coronal contrast-enhanced fat-suppressed T1-weighted image; (**D**,**E**): axial contrast-enhanced fat-suppressed T1-weighted image.

An abdominal phlegmon is an obsolete term that refers to an inflammatory mass that can develop in the setting of penetrating Crohn’s disease. The term phlegmon is ambiguous and relates to an ill-defined inflammatory dense mesenteric mass. It typically spreads without a well-defined wall and involves the mesentery and adjacent bowel with fistula or abscess as possible complication [12]. Inflammatory conglomerate is the accurate terminology to use in the radiological reports. On MRE images, it appears as a variable signal intensity mixed with fat, usually associated with signs of penetrating disease such as complex fistulas [5] (Figure 12, Figure 13 and Figure 14).

**Figure 15:**
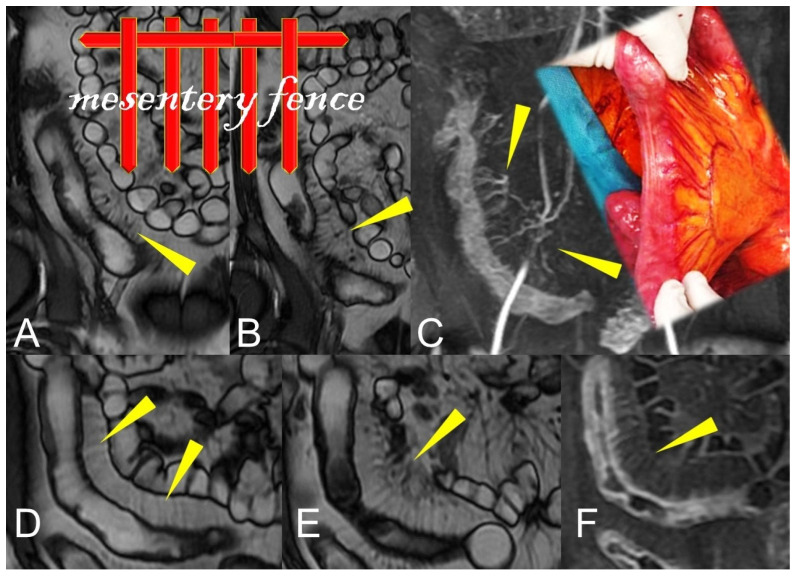
During the active phase, the vascularization of the mesenteric fan increases with an evident engorgement of the vasa recta that takes on the appearance of a clear palisade that stands out against the background of the hypertrophic mesentery. According to some, this aspect commonly resembles the teeth of a hair comb (comb sign) but which we like to imagine instead, due to the thickness of the hypertrophic vasa recta, as a palisade of a fence (mesentery fence). (**A**,**B**,**D**,**E**): Coronal fast imaging employing steady-state acquisition (FIESTA) image, (**C**): coronal contrast-enhanced fat-suppressed T1-weighted maximum intensity projection image, and (**F**): coronal contrast-enhanced fat-suppressed T1-weighted image show engorgement of the vasa recta (yellow arrowheads in (**A**–**F**) images.

Another possible imaging findings of mesenteric inflammation is an abscess that appears as the usual appearance of an encapsulated collection with a rim enhancement and/or a central zone containing air or necrotic material. Moreover, MRE may show perienteric edema/inflammation as a high T2-W signal or a restricted diffusion in the mesenteric fat adjacent to abnormal bowel loops and the engorged vasa recta that supply an inflamed bowel loop (“comb sign”) [6] (Figure 15).

## 3. MRE-Based Indices for the Quantification of Crohn’s Disease Activity and Inflammation

In the last decade, several MRE indices have been proposed to quantify the active disease to grade CD to ensure in any stage the best therapeutic option. They are the Magnetic Resonance Index of Activity (MaRIA), the Clermont score, the Crohn’s Disease Magnetic Resonance Imaging Index (CDMI), the Magnetic Resonance Enterography Global Score (MEGS), and the Lemann index [13]. The most popular and extensively researched MRI grading system for CD is currently the magnetic resonance index of activity (MaRIA) [14,15]. However, considering its complex application, in March 2019 Rimola et al. [16] proposed a simplified magnetic resonance index of activity (MaRIAs) version for an easier and faster assessment of CD’s activity and severity. This index identifies response to therapy with a high level of accuracy; it evaluates the disease’s activity status and it is highly correlated with the simple endoscopic score for Crohn’s disease (SES-CD) and biomarkers [15]. 

The formula of the segmental MARIA is [16]

MARIA = (1 × thickness > 3 mm) + (1 × edema) + (1 × fat stranding) + (2 × ulcers).

A global MaRIA score is the sum of the values calculated in each of the six bowel tracts (the distal ileum, ascending, transverse, descending, sigmoid, colon, and rectum) [14]. 

Simplified MARIA scores greater than 1 identifies segments with active CD with 90% sensitivity and 81% specificity; by contrast, scores of 2 or more detect severe lesions (ulcers) with 85% sensitivity and 92% specificity.

MARIA is a valid and reliable index assessing response to therapy with evidence of correlation with endoscopic mucosal healing (MH), a remarkable endpoint to reach related with the decrease in the number of hospitalizations and surgeries and corticosteroid prescriptions. There is evidence that after treatment, a reduction of 50% or more in the global MARIA score provides good specificity (80%) and sensitivity (73%) for the diagnosis of endoscopic response (reduction in Crohn’s Disease Endoscopic Index of Severity). Similarly, a global MARIA score below 5 shows good specificity (80%) and sensitivity for the diagnosis of endoscopic MH (88%) [16].

Therefore, findings like bowel wall thickening (>3 mm), submucosal edema, fat stranding, superficial or transmural ulcerations are signs of an active disease stage, all present in the MaRIA score. 

Nevertheless, active inflammation is shown on MRE imaging as a layered pattern of enhancement and restricted diffusion [17]. Engorgement of the vasa recta (comb sign) and mesenteric lymphadenopathy, with a typical hyper-enhancement relative to adjacent vessels, also correlates with biologic activity and represents additional findings that improve confidence in a diagnosis of active CD [18].

In the setting of bowel wall evaluation, a stratified/layered pattern of enhancement is a specific radiological sign of active inflammation in CD [17]; on the contrary, homogeneous slow enhancement on delayed post gadolinium T1W images is indicative of inactive or quiescent disease [18,19,20]. The typical “target sign” of active inflammation is a stratified bowel wall appearance due to mucosal and muscle/serosa increased contrast enhancement with intermediate hypo-intensity of edematous submucosa [21].

In chronic disease, there is a fat deposition in the submucosal layer that appears as hypo-intensity in the thickness of the intestinal wall on T2-weighted images with fat saturation (Figure 4B). 

During subacute transmural inflammation, the thickened and fibrotic bowel wall exhibits diffuse and homogeneous contrast enhancement, and the mild mucosal enhancement with hypo-intensity of the deep layers suggests fibrotic condition [19].

In spite of this, stratified or layered enhancement in the intestinal wall, which typically indicates active inflammatory CD, can also exist when fibrostenotic disease coexists with active inflammation. In fact, in patients with small bowel CD, fibrosis and active inflammation sometimes coexist at regions of luminal constriction [18,19,20,21,22].

In this scenario, MRE cine images are helpful to determine the burden of the fibrous component [23]. Cine images can be used to improve the assessment of any undistended loop as well as to evaluate fibrous fixed strictures. Fibrostenotic strictures are usually permanent and fixed; therefore, cine MRE is extremely accurate with respect to the remaining non-dynamic sequences because it documents the lack of distensibility of the affected fibrotic loop [23]. 

Finally, an ileocolonic Crohn’s disease (MRI) Segmental Magnetic Resonance Activity Index (MaRIA) score calculator is available online and can be very useful particularly for those starting out in the field of MRE [24,25].

## 4. Non-Inflammatory Small Bowel Disorders

A large spectrum of non-inflammatory disorders can potentially lead to small bowel mural alterations, including infectious, vascular, or neoplastic conditions. Diagnostic imaging is critical in the assessment and differential diagnosis of small bowel disorders. MRI, especially MRE, allows the evaluation of both small bowel wall and extra visceral structures. 

The correct assessment of the small bowel in MRE necessitates appropriate bowel distension, which can be obtained by administration of the correct amount of a water solution. This solution can be administered orally or through a nasogastric tube [26,27,28].

Most common errors are related to the poor bowel distension, which can be responsible for a false positive, when a small bowel segment is identified as pathological just because it is not very distended, or for a false negative, when the presence of pathology is not recognized in a collapsed segment. Therefore, the knowledge of how to perform an MRE is crucial [22].

Following the MR examination, images are examined to identify pathological small bowel loops. Pathology of the small bowel can occur as thickening of the bowel wall and/or as intraluminal alteration. 

**Figure 16:**
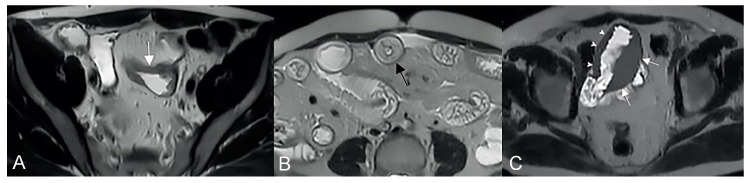
Degree of wall thickening and type of involvement along bowel circumference. (**A**) Axial T2-weighted image shows mild thickening (<1 cm) of an ileal loop in the pelvis (arrow). (**B**) Axial T2-weighted image shows moderate symmetric thickening (>1 cm and <2 cm) of a small bowel loop (black arrow), supported by edema of the submucosal layer. (**C**) Axial T2-weighted image shows marked asymmetric thickening (>2 cm) of an ileal loop in the pelvis (arrows); the other side of the bowel wall is less thick (arrowheads).

In the presence of small bowel thickening, degree, type, and extension of wall thickening must be indicated in the report. Depending on the wall thickness, thickening can be mild (<1 cm), moderate (>1 cm and <2 cm), or marked (>2 cm) [29] (Figure 16A).

**Figure 17:**
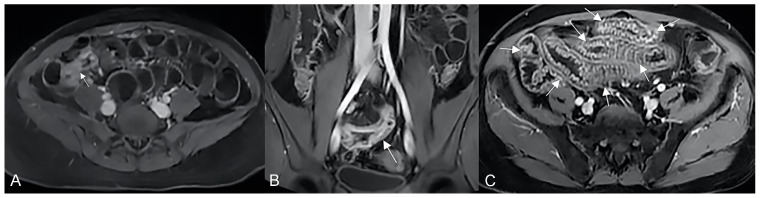
Focal, segmental, or diffuse thickening. (**A**) Axial T1-weighted after gadolinium injection image shows focal thickening of the distal ileum, shorter than 5 cm in length (arrow), with avid contrast enhancement. (**B**) Coronal T1-weighted after gadolinium injection image shows segmental thickening of an ileal loop in the pelvis, with 6–40 cm in length (arrow). (**C**) Axial T1-weighted after gadolinium injection image shows diffuse thickening of a large amount of small bowel loops (>40 cm) (arrows).

Thickening throughout the intestinal loop circumference might be eccentric (asymmetric) or circumferential (symmetric) depending on the type of involvement (Figure 16B,C) [29,30]. According to the length of the pathological bowel tract, thickening can be focal, segmental, or diffuse. Bowel thickening is defined as focal when the pathological tract is shorter than 5 cm in length (Figure 17A); segmental thickening refers to 6–40 cm in length of thickened small bowel (Figure 17B), while it is considered diffuse when a large amount of small bowel loops (>40 cm) is affected (Figure 17C) [29,30]. After contrast-medium injection, various patterns of wall enhancement can be described: stratified, white, and gray [30]. Contrast enhancement is classified as stratified when there is hyperintensity of the inner layer (muco-sa), hypointensity of the intermediate layer (submucosa), and hyperintensity of the outer layer (muscle and serosa layers); this is caused by mucosal hyperemia and submucosal edema. If stratified contrast enhancement is associated to fat deposits in the submucosal layer, it is called “fatty halo” sign [30,31]. 

The white pattern is caused by intestinal wall enhancement that is more than or equal to vein enhancement of the same degree. The gray pattern (diminished or mild enhancement of the bowel wall) occurs when the enhancement of the bowel wall is similar to muscle attenuation on a contrast-enhanced exam [30].

MRI is also very significant in identifying intraluminal alterations thanks to the possibility of matching imaging morphological information with functional data, the high contrast resolution, and a relatively safe intravenous injection of gadolinium. The elevated signal intensity of the intraluminal content on balanced steady-state GRE and echo-planar fast spin echo sequences allows the identification of the intraluminal alterations showing intermediate signal intensity [32].

**Figure 18:**
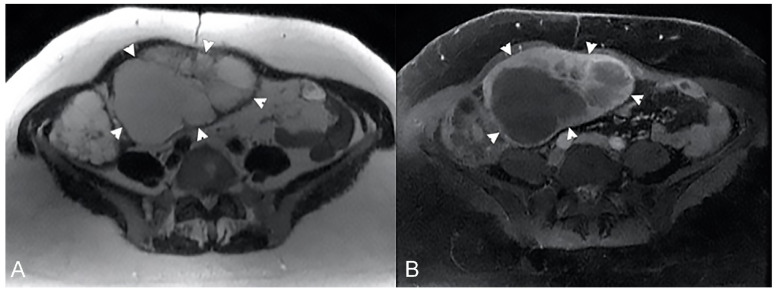
Example of marked thickening due to a malignant condition. (**A**) Axial T2-weighted and (**B**) axial T1-weighted after gadolinium injection images show a marked, focal, and asymmetrical small bowel thickening, with inhomogeneous contrast enhancement (arrowheads), caused by a malignant condition.

Once bowel pathology has been identified, the radiologist’s first step is trying to define the benign or malignant nature of the alteration. When intestinal pathology is found, the radiologist’s initial step is to determine if the alteration is benign or malignant, starting from patient’s history and clinical information. In the presence of small bowel thickening, a benign condition is probable when thickening is mild or moderate in degree, focal or segmental and symmetrical (Figure 17A,B), with homogeneous or stratified contrast enhancement; on the other hand, a malignancy is probable when there is a significant mural thickening, focal and asymmetrical, with inhomogeneous contrast enhancement (Figure 18) [29].

**Figure 19:**
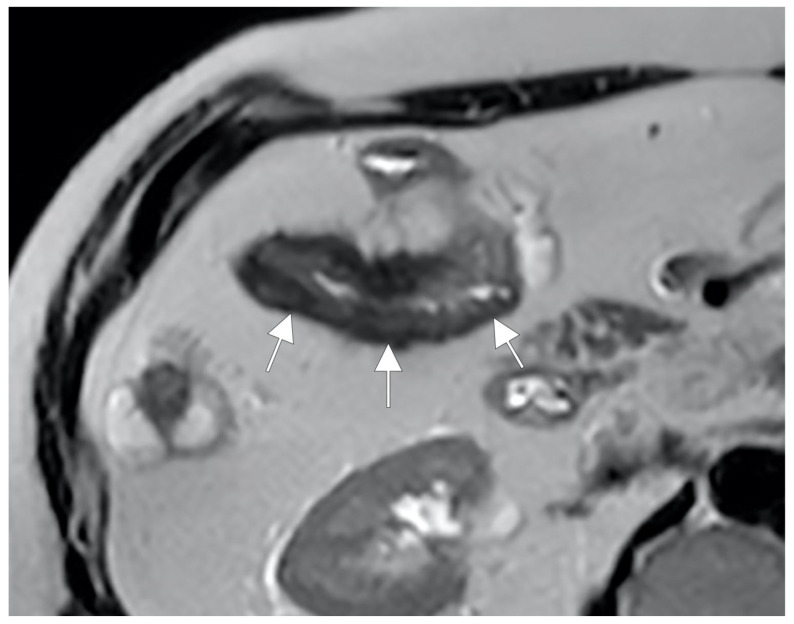
Example of marked thickening due to a benign condition. Axial T2-weighted image shows marked segmental thickening of a small bowel loop in the right abdominal quadrant (arrows), due to a benign condition (Crohn’s disease).

Diffuse thickening, especially when almost all small bowel loops are affected (Figure 17C), is always due to benign diseases such as infections, inflammation, and parietal edema, as observed in hypoproteinemia or mesenteric ischemia. Sometimes, marked thickening can be also noticed in benign diseases, such as severe infections (Figure 19) [29].

In case of intraluminal alteration, filling defect with low signal intensity on FIESTA sequences and high enhancement similar to the bowel mucosa after intravenous gadolinium injection can be detected [32]. However, superficial mucosal ulcerations are usually not well detected by MRI despite correct bowel distention [4].

After determining whether the results are benign or malignant, the radiologist must attempt to make a conclusive diagnosis by correlating MRI with clinical and laboratory data.

## 5. Small Bowel Tumors

Despite accounting for over 75% of the length and 90% of the mucosal surface area of the gastrointestinal tract, small bowel neoplasms are uncommon, accounting for 3% to 6% of all gastrointestinal tumors. They can be divided into benign and malignant. Benign cancers include inflammatory polyps, adenomas, leiomyomas, and lipomas. Neuroendocrine tumors, gastrointestinal stromal tumors, lymphomas, adenocarcinomas, and metastases are examples of malignant malignancies. 

Tumors appears as nodules, annular lesions, excavating masses, or stricturing lesions. 

Patients with small bowel tumors may complain of various symptoms such as anemia, weight loss, gastrointestinal bleeding, generalized or localized abdominal pain, bowel obstruction or perforation [33,34].

The main MR features of small bowel tumors are summarized in Table 1.

### 5.1. Leiomyoma

Among benign tumors of the small bowel, leiomyomas are the most common symptomatic forms. They are preferentially located in the stomach, developing in the circular or longitudinal muscle layer of the bowel wall [34,35]. Leiomyomas are often asymptomatic and slow growing; an unclear abdominal discomfort due to ulceration or partial obstruction may sometimes be present. The most common sign is the gastrointestinal hemorrhage manifesting as hematemesis, melena or unexplained anemia.

Histological diagnosis is made when positivity for smooth muscle myosin heavy chain and negativity for CD117 (c-kit) and S-100 with proliferation index (Ki-67) < 10% are observed in the specimen. Based on the type of growth, four types of leiomyoma can be identified: submucosal or intraluminal (the most frequent), intramural, subserosal or extraluminal, and bidirectional (dumbbell-shaped) [34].

On MRI, leiomyomas appear as a spherical or ovoid nodule, ranging from 1 to 10 cm in diameter, with soft tissue intensity and uniform enhancement after gadolinium injection. Differential diagnosis with leiomyosarcoma can be possible when there are signs of benignity, such as homogeneous enhancement after contrast-medium injection and absence of mesenteric alterations or metastases [33,34,35].

### 5.2. Adenoma

Adenomas are the most prevalent asymptomatic benign small bowel tumors, accounting for 14–20% of all benign small bowel neoplasms. They are composed of glandular epithelium with a specific malignant predisposition, are identical to their large bowel counterparts (tubular, villous, tubulovillous), and can be premalignant. The duodenum is the most prevalent location for adenomas [36]. Adenomas can be asymptomatic or responsible for non-specific symptoms, such as vague abdominal pain or abdominal discomfort; sometimes the acute presentation may be due to bowel obstruction or intussusception [37].

Adenomas have an epithelial origin, with a dysplastic or irregular columnar surface epithelium and lamina propria, forming more elongated, straight frondlike or villous projections that originate from the mucosal base. On MRI, they appear as sessile or pedunculate well-defined, soft tissue mass surrounded by a thin rim of oral contrast that shows moderate enhancement after intravenous contrast administration. They may exhibit bleeding, obstruction, and intussusception. MR images can help to differentiate adenomas from adenocarcinomas by identifying smooth margins, lack of mesenteric invasion, and clear fat planes around the tumor [38].

### 5.3. Neuroendocrine Tumors (NET)

Among malignant cancers of the small intestine, neuroendocrine tumors are the most common, arising from enterochromaffin cells, and there is an equal distribution between men and women, with an average age of onset of 65 years [34,35,36,37,38,39]. In the past, the term “carcinoid” was also used to indicate “neuroendocrine tumor”. Nowadays, “neuroendocrine tumor” (NET) is the only recommended term, and it includes well-differentiated NET and poorly differentiated neuroendocrine carcinoma (NEC) [39,40]. The most frequent localization in the small bowel is the ileum.

Even if abdominal pain, weight loss, melena, intestinal obstruction, or ischemia are present, NETs can be asymptomatic. An accurate diagnosis is also based on the overproduction of circulating biologically active hormones, peptides and amines, such as gastrin, glucagon, insulin, serotonin, somatostatin, vasoactive intestinal polypeptide, CgA, and 5-HIAA. 

**Figure 20:**
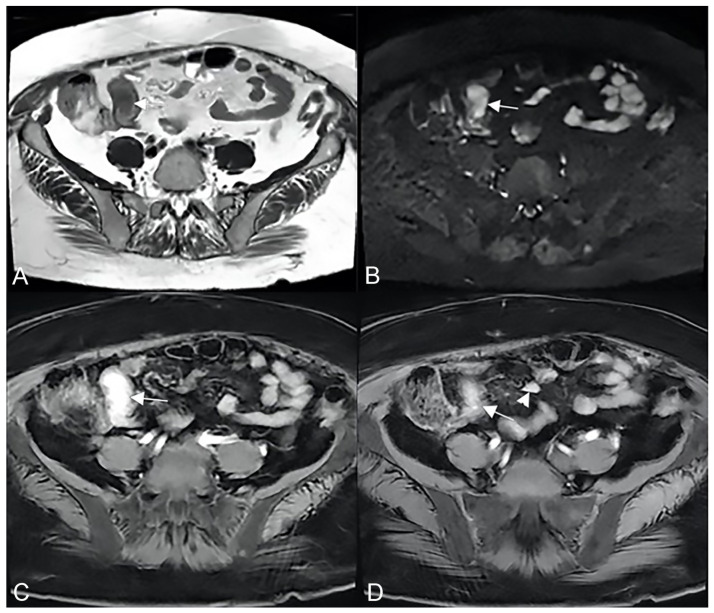
Example of NET in a 56-year-old female patient with abdominal pain and weight loss. (**A**) Axial T2-weighted, (**B**) DWI, and (**C**,**D**) T1-weighted after gadolinium injection images show focal asymmetric wall thickening of the distal ileum (arrow), with restriction in DWI (arrow in (**B**)) and marked enhancement in the arterial phase (arrow in (**C**,**D**)); an hypervascular mesenteric enlarged lymph node is also evident (arrowhead in (**D**)).

NET may present on MRI as a tiny submucosal nodule or as focal asymmetric wall thickening, with marked enhancement in the arterial phase, sometimes with hyper-vascular mesenteric enlarged lymph nodes (Figure 20). Sometimes a focal bowel wall thickening without evidence of a nodular lesion can be appreciated for the desmoplastic reaction, typical of this type of cancer. Sometimes evidence of metastases without detection of the primary tumor may occur [34,35,36,37,38,39].

Other malignant disorders of the small bowel should be considered in the differential diagnosis. NET diagnosis is made when avid enhancement after gadolinium injection, desmoplastic reaction, and hyper-vascular enlarged lymph nodes are observed. NETs can become complicated with obstruction due to the desmoplastic reaction or involvement of serosa [39].

### 5.4. Gastrointestinal Stromal Tumors (GIST)

They include benign and malignant forms, representing the most common mesenchymal cancer of the gastrointestinal system. People over 40 years are more frequently involved [33,34,41].

GISTs arise from interstitial cells of Cajal. It is distinguished from leiomyomas by the frequent expression of a particular tyrosine kinase growth factor receptor (c-KIT) [33,34,41]. Common symptoms can be anemia and upper gastrointestinal bleedings, abdominal pain or discomfort, and a palpable mass; small GISTs are usually asymptomatic.

If there are some diagnostic doubts (especially in CD117 immuno-negative suspected GIST), immuno-hystological diagnosis requires CD117 immunopositivity or molecular analysis for activating mutations in KIT or PDGFRA.

**Figure 21:**
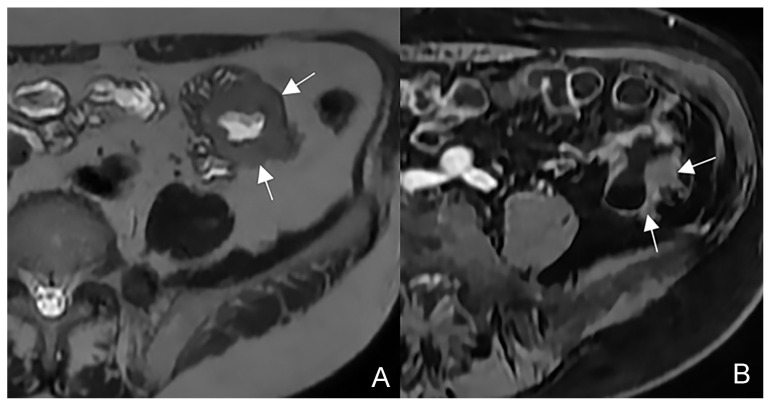
Example of GIST in an old man with abdominal discomfort and a palpable mass. (**A**) Axial T2-weighted and (**B**) axial T1-weighted after gadolinium injection images show an asymmetrical mass-like thickening of a jejunal loop (arrows) with aneurysmal pattern.

On MRI, GISTs usually look like asymmetrical mass-like thickenings of the bowel wall; the malignant form often shows inhomogeneous enhancement after gadolinium injection due to necrotic or bleeding or cystic findings (Figure 21) [33,34,41].

Unlike adenocarcinoma, exophytic growth with infiltration of the muscular layer and asymmetrical thickening of the bowel wall characterize this type of cancer; therefore, bowel occlusion rarely occurs even when it reaches large dimensions [33]. Like lymphomas, GISTs can also be characterized by aneurysmal dilation of the small bowel. A diameter wider than 10 cm, local infiltration, and metastatic involvement of liver, peritoneum and omentum are indicative of malignancy.

### 5.5. Lymphoma

Lymphomas are the third most common cancer of the small bowel. Ileum is involved in more than 60% of all cases because of the lymphoid tissue more contained in the terminal ileum than in the duodenum and the jejunum [34,42,43,44].

Lymphomas can involve the bowel primarily or consequently to systemic disease.

Epigastric discomfort, weight loss, and anorexia are common symptoms. Nausea and vomiting are more common in the later stages of the disease. The small bowel is primarily affected by bleeding, the presence of an abdominal tumor, and bowel perforation.

Endoscopic biopsies are required because laboratory tests may reveal low hemoglobin levels, pancytopenia, and elevated tumor markers such as LDH or CRP lactate dehydrogenase. Primary lymphomas may appear in the polypoid, mass, infiltrative, or aneurysmal form.

The polypoid pattern is so called for the evidence of polypoid nodules, without thickening of the bowel wall; in this form, intestinal intussusception could be a frequent complication. 

The mass pattern refers to the evidence of a mushroom-shaped mass with exophytic extension, responsible for complications such as ulceration and fistulas.

**Figure 22:**
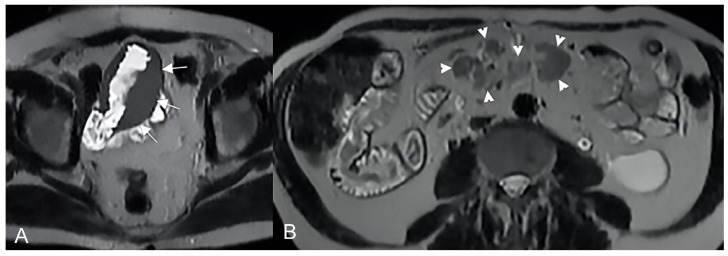
Example of Lymphoma in a 71-year-old male patient (the same patient in Figure 1C). (**A**) Axial T2-weighted image shows a marked asymmetric thickening of an ileal loop in the pelvis (arrows). (**B**) An axial T2-weighted image on a different plane shows multiple enlarged mesenteric lymph nodes (arrowheads).

The infiltrative pattern is characterized by circumferential involvement of the wall, and it is responsible for different degrees of wall thickening and disappearing of the normal wall layers. Bowel wall infiltration looks like segmental and circumferential thickening, with homogeneous enhancement after gadolinium injection [33,34] (Figure 22).

The aneurysmal pattern often coexists with the infiltrative one and it can be its evolution. In this pattern, lymphomatous infiltration and disruption of the muscle layer and neural plexuses may be appreciated, resulting in loss of muscle tone and consequent bowel dilatation. 

Regarding the differential diagnosis, an aggressive adenocarcinoma with ulcerations should be considered.

MRI is suggestive for lymphoma when a bowel wall aneurysmal mass without proximal bowel occlusion is seen. The diagnosis can be supported by splenomegaly and retroperitoneal and mesenteric enlarged lymph nodes. Adenocarcinoma is characterized by the invasion of the surrounding mesenteric folds (less for lymphoma), while mesenteric ill-defined confluent masses enveloping the bowel are more frequent in lymphomas [33].

### 5.6. Adenocarcinomas

Adenocarcinomas account for around 2% of all gastrointestinal tumors and are the most prevalent type of small bowel primary malignancy.

Adenocarcinomas typically develop from the glandular epithelium, consisting of villous or tubular structures. Depending on gland formation and mucin production, they can be classified into mucinous, signet-ring cell, and undifferentiated forms, even if moderately to well-differentiated forms are more frequent.

The site most frequently involved is the duodenum (50% of cases), followed by the jejunum and ileum. Patients with adenocarcinomas frequently have altered bowel habits (constipation and/or diarrhea) and iron deficiency anemia. Among laboratory tests, a high level of CEA is most probably associated with adenocarcinomas. Adenocarcinoma may have different patterns such as infiltrating, stenosing, or polypoid [45]. 

**Figure 23:**
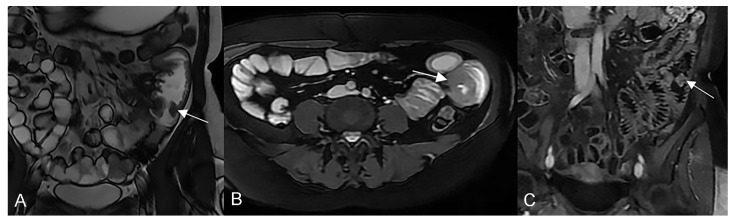
Example of adenocarcinoma in a middle-aged man. (**A**) Coronal T2-weighted, (**B**) axial T2-weighted fat-suppressed, and (**C**) coronal T1-weighted after gadolinium injection images show a focal irregular thickening of a jejunal loop (arrow) with inhomogeneous enhancement after gadolinium injection (arrow in (**C**)).

The polypoid pattern is more frequently observed in the duodenum, while stenosing and infiltrating patterns are generally located in the jejunum or ileum due to circumferential infiltration of the wall (Figure 23). Full wall infiltration, serosal involvement, and extension into the adjacent mesenteric tissue are common signs that are typically seen. 

MRI typically displays inhomogeneous signal intensity and moderate enhancement. Lymph nodal involvement is not of the same entity as lymphomas. MRI may be required to identify and stage this condition by detecting hepatic, lymph nodal, and peritoneal involvement. Sometimes, adenocarcinoma of the ileum can simulate Crohn’s disease. However, the suspicion of malignancy arises from the absence of local hypervascularization and the evidence of a single finding without skip lesions [6].

### 5.7. Leiomyosarcoma 

This cancer arises from the smooth muscle of the bowel wall, and it is usually slow growing with prevalent extraluminal and eccentric pattern.

In the early stages, leiomyosarcomas are often asymptomatic. Fatigue, fever, weight loss, a general sensation of ill health (malaise), and nausea and vomiting may occur in the later stages. Another frequent symptom is bleeding in the gastrointestinal system, causing black, tarry, foul-smelling stools (melena), or blood vomiting (hematemesis). 

Laboratory tests may reveal low hemoglobin levels and neutropenia. 

On MRI, it looks like a large inhomogeneous mass with a necrotic and/or hemorrhagic core. The signs of malignity, such as large dimensions (>5 cm), inhomogeneous enhancement after gadolinium injection, and evidence of mesenteric or metastatic disease, help in the differential diagnosis with leiomyoma [33,34].

### 5.8. Metastasis 

Metastases account for about 50% of all small bowel tumors and they should be considered as the most probable diagnosis in an oncological patient. 

There are four ways of metastasizing a primary cancer into the small bowel: direct invasion, intraperitoneal, lymphatic, and hematogenous spread. The tumors originating from the gastrointestinal system metastasize more frequently to the small bowel; moreover, melanomas, lung cancers, breast cancers, and thyroid tumors should be considered [46].

Small bowel metastases are relatively rare. In general, non-specific clinical manifestations such as intermittent stomach pain, nausea, vomiting, and weight loss are prevalent.

Laboratory tests may reveal iron deficiency anemia and thrombocytopenia.

The MR aspect of metastases may be variable, appearing as smooth or rounded polypoid nodules that can cause ulceration and invagination, or as wall thickening with invasion of adjacent mesenteric tissue, particularly in cases of mucinous origin. They are often focused on the anti-mesenteric side of the bowel [34,47,48]. 

## 6. Other Diseases

Different non-inflammatory conditions are associated with small bowel wall thickening, such as tuberculosis, ischemia, vasculitis, radiation enteritis, hemorrhage, and amyloidosis.

### 6.1. Small Bowel Tuberculosis

In rare cases, tuberculosis can affect small bowel, especially ileo-cecal segment. 

Fever, anorexia, weight loss, stomach pain, abnormal bowel habits, recurrent partial bowel obstruction, or the existence of an abdominal tumor could be clinical symptoms. Extra-intestinal symptoms such as polyarthritis, uveitis, and (less commonly) erythema nodosum may be associated.

The tuberculin skin test (Mantoux test) with an induration of 20 or greater is the primary laboratory test for tuberculosis diagnosis. 

**Figure 24:**
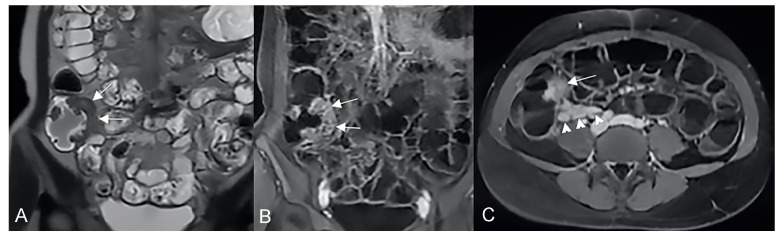
A case of ileal tuberculosis in a 34-year-old female patient. (**A**) Coronal T2-weighted image and (**B**,**C**) T1-weighted after gadolinium injection images in coronal and axial planes show thickening of the distal ileum and ileocecal valve (arrows). Enlarged lymph nodes are also observed in the adjacent mesentery (arrowheads).

In the first phase of the disease, MRI shows a circumferential and symmetrical wall thickening involving the terminal ileum and the cecum (Figure 24), while the ileocecal valve and the cecum may show an asymmetrical wall thickening in a later stage. Enlarged lymph nodes with necrosis are also observed [29,30].

Diagnosis of bowel tuberculosis can be suggested when thoracic findings and/or other abdominal signs of tuberculosis, such as hepato-splenic involvement and peritoneal dissemination, are observed.

Nowadays, computed tomography (CT) is used as a first line imaging modality for differentiating small bowel tuberculosis from Crohn’s disease. MRI is preferred in pediatric patients and for follow-up studies [49].

### 6.2. Ischemia 

Small bowel ischemia is caused by different pathological entities. 

#### 6.2.1. Acute Ischemia

Acute ischemia can be induced by the occlusion of the superior mesenteric artery (about 50% of the time), caused by an embolus usually starting from the heart or thrombosis of the mesenteric artery (about 25% of the time), and caused by atherosclerotic stenosis. Other causes are non-occlusive mesenteric ischemia (about 20% of the time), due to a reduction in the bowel blood supply with secondary mesenteric vasoconstriction or thrombosis of mesenteric vein (about 5% of the time).

Severe stomach pain (in the periumbilical area or the right umbilical fossa), nausea, vomiting, diarrhea, and blood per rectum are possible clinical symptoms.

Leukocytosis, neutrophilia, a shift to immature leukocytes in the differential, and metabolic acidosis could all be detected by laboratory tests.

**Figure 25:**
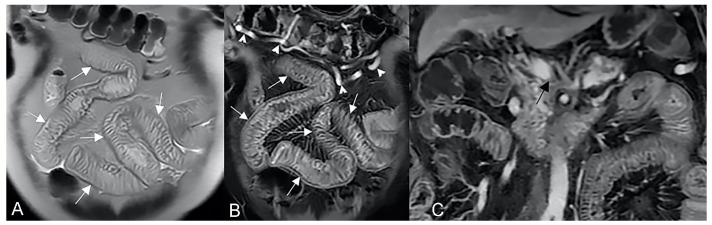
A case of small bowel ischemia in a 61-year-old male patient. (**A**) Coronal T2-weighted and (**B**,**C**) coronal T1-weighted after gadolinium injection images show diffuse thickening of small bowel loops (white arrows) with stratified contrast enhancement due to thrombosis of superior mesenteric vein (black arrow in (**C**)). Multiple collateral veins (arrowheads in (**B**)) and free abdominal fluid are also observed.

Extension, degree of wall thickening, and type of wall enhancement depend on the cause of ischemia (occlusion of the artery, occlusion of a vein or hypoperfusion), the degree of ischemia (transient or durable), and the coexistence of infection or hemorrhage. On MRI, bowel wall thickening is usually present, even if this sign is not observed in arterial acute occlusion. Acute ischemia can simulate different bowel conditions, and the pattern of wall thickening could be localized or diffuse, focal, or segmental, superficial or transmural. In the early stages of bowel ischemia, a stratified contrast enhancement can be observed, showing mucosal hyperemia or hypoperfusion and submucosal edema (Figure 25). Other findings such as occlusion of the mesenteric artery or vein, mesenteric venous engorgement, mesenteric edema, bowel distension, and ascites can be associate [50].

Severe ischemia is characterized by the bowel pneumatosis with the coexistence of gas in the portal vein. Pneumatosis is the presence of air bubbles in the bowel wall, located in submucosal or subserosal layers. These bubbles are more apparent on gradient-echo images due to “blooming” associated with magnetic field inhomogeneities at air-tissue interfaces [51]. Necrosis is usually responsible for a thin aspect of the bowel wall [52].

#### 6.2.2. Chronic Ischemia

Chronic mesenteric ischemia is caused by insufficient blood flow in the postprandial period. It is usually caused by an atherosclerotic occlusive condition that affects the origin of the mesenteric arteries [53].

Clinical symptoms may include stomach postprandial pain, food avoidance in later stages, weight loss, diarrhea, nausea, and fever. 

Laboratory tests do not help with reaching a diagnosis.

Diagnostic imaging can identify mesenteric arterial stenosis; recently, some authors demonstrated the value of mesenteric blood flow measurement with contrast-enhanced MRI, resulting in a significant lower increase in the postprandial period in patients with chronic mesenteric ischemia [54]. Other authors studied mesenteric hemodynamics before a meal and in the postprandial period by using the 4D flow MRI, demonstrating a significant difference in the redistribution of blood flow [55].

#### 6.2.3. Vasculitis 

Vasculitis represents a rare cause of bowel ischemia that occurs predominantly in the polyarteritis nodosa, typical of young patients. MRI usually shows bowel wall thickening with stratified enhancement, involving unusual tracts (stomach, duodenum, and rectum) and without confinement in a vascular territory. The distinction between vasculitis-related ischemia and other causes of mesenteric ischemia can be difficult to discern based on the imaging findings alone, so association with clinical factors (young age, fever, weakness, myalgia, headache) is mandatory to make a correct diagnosis [29,30]. 

### 6.3. Radiation Enteritis 

Due to its disposition in the abdomen, the small bowel is particularly sensitive to abdominal or pelvic radiation therapy, especially when it is preceded by pelvic surgery (i.e., hysterectomy or proctectomy) [56].

In the early stages, the mucosal damage leads to cellular change from normal villous epithelium to non-functioning cells, losing the barrier effect. In a later phase, the damage is due to the association of submucosal fibrosis and vascular degeneration [56].

Anorexia, nausea, vomiting, stomach cramping, and diarrhea are common early symptoms. A partial obstruction of the small bowel could be a late sign.

Laboratory testing can reveal low hemoglobin levels, high white blood cell counts due to intestinal perforation or necrosis, and blood in stool investigations.

**Figure 26:**
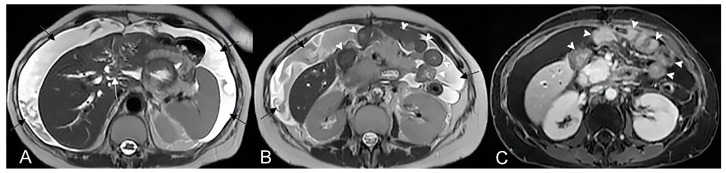
A case of radiation enteritis in a 54-year-old female patient with Klatskin tumor. (**A**) Axial T2-weighted image shows an intrahepatic Klatskin tumor (arrow), with dilatation of the intrahepatic bile ducts (asterisks). (**B**) Axial T2-weighted and (**C**) axial T1-weighted after gadolinium injection images on a different plane display diffuse thickening of upper small bowel loops (arrowheads), due to radiation therapy for Klatskin tumor. A massive amount of free abdominal fluid is also evident (black arrows in (**A**,**B**)).

If radiation enteritis is suspected, the diagnosis must be confirmed by imaging, for its ability to identify the loops involved, to classify the pattern (segmental or diffuse), and to search for complications, especially if clinicians consider the possibility of a surgical treatment [56]. In case of acute radiation enteritis, MRI shows diffuse mild segmental wall thickening in combination with mesenteric fat stranding (Figure 26); the distal ileum is usually the most frequently affected site [29]. The most probable radiological sign in chronic radiation enteritis is wall submucosal thickening. Other chronic radiological signs are bowel wall thickening with strictures, obstruction, and fistula [29].

### 6.4. Eosinophilic Enteritis

This type of disorder is characterized by the pathological accumulation of eosinophils in the bowel wall, without evidence of other conditions of eosinophilia (for example, malignancy, parasitic infections, and drug reactions) [57].

Eosinophils may infiltrate the mucosal layer, the muscular layer, or the serosal layer of the bowel wall, with a predilection for the distal antrum and jejunal loops, so symptoms depend on which layer is involved.

Clinical manifestations consist in nonspecific symptoms, so diagnosis requires pathological evidence of eosinophilic infiltration of at least one intestinal tract and exclusion of other causes of enteric eosinophilia.

**Figure 27:**
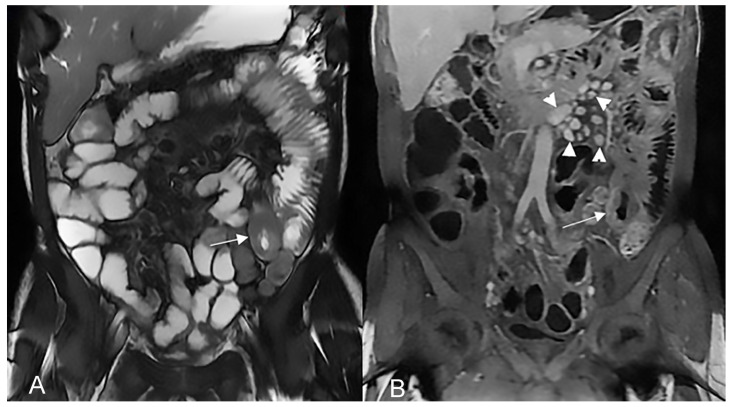
An example of eosinophilic enteritis. (**A**) Coronal T2-weighted fat-suppressed and (**B**) coronal T1-weighted after gadolinium injection images show focal thickening of a jejunal loop (arrow) with multiple enlarged lymph nodes in the mesentery (arrowheads in (**B**)).

MRI demonstrates bowel wall thickening with abnormal enhancement. In the mucosal form, imaging shows ulcers and polyps, while when the muscle layer is involved, imaging may show intestinal strictures and reduced luminal caliper (Figure 27). When the serosal layer is infiltrated, pathological enhancement of serosa, omental thickening, ascite, and pleural effusion can be observed; mesenteric lymphadenopathy with central necrosis may also be observed [58].

## 7. Conclusions

Small bowel disorders present a significant challenge for the radiologist. When performed correctly, MRI is a useful tool for diagnosing small bowel disorders that manifest as mural thickening or intraluminal alteration. An attempt to formulate a reliable diagnosis should be made once the benign or malignant nature has been determined. Furthermore, it is critical to link imaging findings with clinical and laboratory data in order to develop the fewest and most accurate diagnostic hypothesis. 

## Figures and Tables

**Table 1:** MR signal intensity of small bowel tumors.

Bowel Tumors	T1 Signal Intensity	T2 Signal Intensity	T1 Contrast-Enhanced Signal Intensity	Most Frequent Site
Leiomyoma	Low	Low	High and Homogeneous	ileum
Adenoma	Low	High	High and Homogeneous	jejunum
Neuroendocrine tumor	Low	Low	High and Homogeneous	ileum
Gastrointestinal stromal tumor	Low	High	High and inhomogeneous	jejunum
Lymphoma	Low	Low	Mild and inhomogeneous	ileum
Adenocarcinoma	Low	Low	Mild and inhomogeneous	jejunum
Leiomyosarcoma	Low	Low	Mild and inhomogeneous	ileum

## Data Availability

Data are available from the authors upon request (to Savino Cilla or Antonio Pierro) for researchers of academic institutes who meet the criteria for access to the confidential data.
